# The Anti-Atherosclerotic Effect of Naringin Is Associated with Reduced Expressions of Cell Adhesion Molecules and Chemokines through NF-κB Pathway

**DOI:** 10.3390/molecules21020195

**Published:** 2016-02-05

**Authors:** Tun-Pin Hsueh, Jer-Ming Sheen, Jong-Hwei S. Pang, Kuo-Wei Bi, Chao-Chun Huang, Hsiao-Ting Wu, Sheng-Teng Huang

**Affiliations:** 1 Department of Chinese Medicine and Mitochondrial Research Unit, Kaohsiung Chang Gung Memorial Hospital and Chang Gung University College of Medicine, No. 123, Da-Pei Road, Niao-Sung District, Kaohsiung 83301, Taiwan; melaopin@gmail.com (T.-P.H.); r12042@cgmh.org.tw (J.-M.S.); bikw@adm.cgmh.org.tw (K.-W.B.); b8606059@tmu.edu.tw (H.-T.W.); 2 Graduate Institute of Clinical Medical Sciences, Chang Gung University, No. 259, Wen-Hua 1st Road, Kwei-Shan, Tao-Yuan 33302, Taiwan; jonghwei@mail.cgu.edu.tw; 3 Division of General Surgery, Ministry of Health and Welfare Pingtung Hospital, No. 270, Ziyou Road, Pingtung City, Pingtung County 900, Taiwan; hcj5311@yahoo.com.tw; 4 School of Chinese Medicine, China Medical University, No. 91, Hsueh-Shih Road, Taichung 40402, Taiwan

**Keywords:** naringin, atherosclerosis, TNF-α, chemokines, NF-κB

## Abstract

Naringin has been reported to have an anti-atherosclerosis effect but the underlying mechanism is not fully understood. The aim of this study is to investigate the impact of naringin on the TNF-α-induced expressions of cell adhesion molecules, chemokines and NF-κB signaling pathway in human umbilical vein endothelial cells (HUVECs). The experiments revealed that naringin, at concentrations without cytotoxicity, dose-dependently inhibited the adhesion of THP-1 monocytes to the TNF-α-stimulated HUVECs. The TNF-α-induced expressions of cell adhesion molecules, including VCAM-1, ICAM-1 and E-selectin, at both the mRNA and protein levels, were significantly suppressed by naringin in a dose dependent manner. In addition, the TNF-α-induced mRNA and protein levels of chemokines, including fractalkine/CX3CL1, MCP-1 and RANTES, were also reduced by naringin. Naringin significantly inhibited TNF-α-induced nuclear translocation of NF-κB, which resulted from the inhibited phosphorylation of IKKα/β, IκB-α and NF-κB. Altogether, we proposed that naringin modulated TNF-α-induced expressions of cell adhesion molecules and chemokines through the inhibition of TNF-α-induced activation of IKK/NF-κB signaling pathway to exert the anti-atherosclerotic effect.

## 1. Introduction

Atherosclerosis is the major reason for cardiovascular disease (CVD) and one of the leading causes of death worldwide [1]. It is caused by fibro-fatty plaques that protrude into vascular lumen and weaken the underlying media. Atherosclerotic plaques form in arteries and are characterized by inflammation, lipid and macrophage accumulation, cell death, and fibrosis. Its complex mechanism involves blood leukocytes, endothelial cells, vascular smooth muscle cells, and cytokines in the arterial intima [2]. It is implicated in the formation of early fatty streaks when the endothelium is activated and expresses chemokines and adhesion molecules leading to monocyte/lymphocyte recruitment and infiltration into the subendothelium. Adhesion molecules and chemokines, including endothelial selectin (E-selectin), vascular cell adhesion molecule-1 (VCAM-1), intracellular adhesion molecule-1 (ICAM-1), fractalkine, interleukine-8 (IL-8), and monocyte chemotactic protein-1 (MCP-1), regulated on activation of normal T cell expressed and secreted (RANTES), are the crucial pathogenic elements in atherosclerosis and have been reported to be up-regulated in the cells of atherosclerotic lesions [3,4]. The up-regulation of these molecules may influence growth factor production and medial smooth muscle cell migration [5]. Inflammatory cytokines and oxidative stress also accelerate the development of atherosclerosis in patients with diabetes or animal models of diabetes [6,7,8]. NF-κB is considered an inflammatory marker that is associated with various inflammatory diseases. It has been demonstrated that NF-κB activation is the underlying molecular mechanism for constitutive expression of adhesion molecules and chemokines on human atherosclerosis sections [9]. Therefore, blocking of the increased production and/or biological activity of pro-inflammatory mediators including adhesion molecules and chemokines may have therapeutic benefits for CVD.

Flavonoids are natural substances with variable phenolic structures. They can be found in fruits, vegetables, grains, bark, roots, stems, flowers, tea, and wine. Flavonoids have received much attention in the literature over the past decades. They have been demonstrated to exert anti-atherosclerotic effects, anti-thrombogenic effects, anti-inflammatory effects, anti-oxidative effects, anti-tumor effects, anti-osteoporotic effects, and anti-viral effects [10]. They prevent development of atherosclerosis and reduce endothelial injury to decrease cardiovascular morbidity and blood pressure in humans [11,12,13]. Various clinical studies have indicated that flavonoids may have an inverse association with cardiovascular events [14,15,16].

Naringin is a major compound of flavanone and can be extracted from Chinese medicines such as *Rhizoma drynariae* (GuSuiBu), *Fructus Aurantii immaturus* (Zhi Shi), and red tangerine peel. Studies have indicated that naringin has a protective function against atherosclerosis. A study proposed that naringin was closely involved with decreased hepatic acyl-CoA: cholesterol acyltransferase (ACAT) activity, as well as the down-regulation of vascular cell adhesion molecule-1 (VCAM-1) and monocyte chemotactic protein-1 (MCP-1) genes [17]. Another study reported that naringin significantly decreased both fatty streak formations in the thoracic aorta and neointimal macrophage infiltration on hypercholesterolemic rabbits [18].

Therefore, it was suggested that the anti-atherosclerotic effect of naringin was mediated by inhibition of ICAM-1 expression, which leads to inhibition of macrophage infiltration. However, the mechanisms of the anti-atherogenic effects of naringin in atherosclerotic plaque formation have never been studied in detail. The aim of this study was to analyze the effects of naringin on tumor necrosis factor alpha (TNF-α)-induced signaling such as the cell adhesion molecules and chemokines in human umbilical vein endothelial cells (HUVECs).

## 2. Results

### 2.1. No Cytotoxic Effect of Naringin on HUVECs

**Figure 1:**
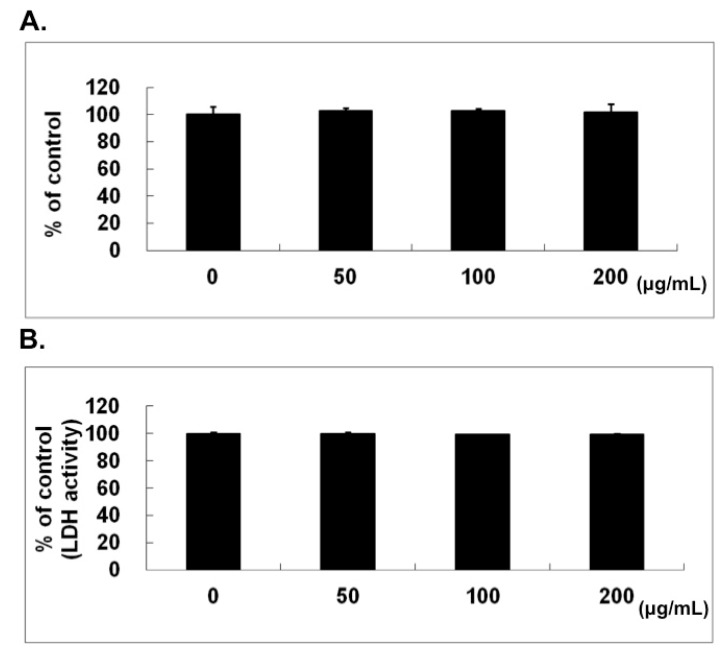
No cytotoxic effect of naringin on human umbilical vein endothelial cells (HUVECs). (**A**) The cell viability was determined by standard 3-(4,5-dimethylthiazol-2-yl)-2,5-diphenyltetrazolium bromide (MTT) assay with different concentrations of Naringin for 24 h; (**B**) The cytotoxic effect of Naringin was evaluated by the lactate dehydrogenase (LDH) assay using conditioned medium collected from HUVECs and treated with different concentrations of Naringin for 24 h.

As determined by 3-(4,5-dimethylthiazol-2-yl)-2,5-diphenyltetrazolium bromide (MTT) assay, the cell viability of HUVECs was not inhibited by naringin at concentrations up to 200 μg/mL for 24 h (Figure 1A). The lactate dehydrogenase (LDH) activity measured in conditioned medium also demonstrated no cytotoxic activity, as shown in Figure 1B. The phase-contrast microscopic examination (100×) on the cell morphology of HUVECs after naringin treatment did not reveal any obvious change and there was no sign of apoptosis and/or necrosis (data not shown).

### 2.2. Naringin Inhibited the Adhesion of Monocytes to TNF-α-Stimulated HUVECs

**Figure 2:**
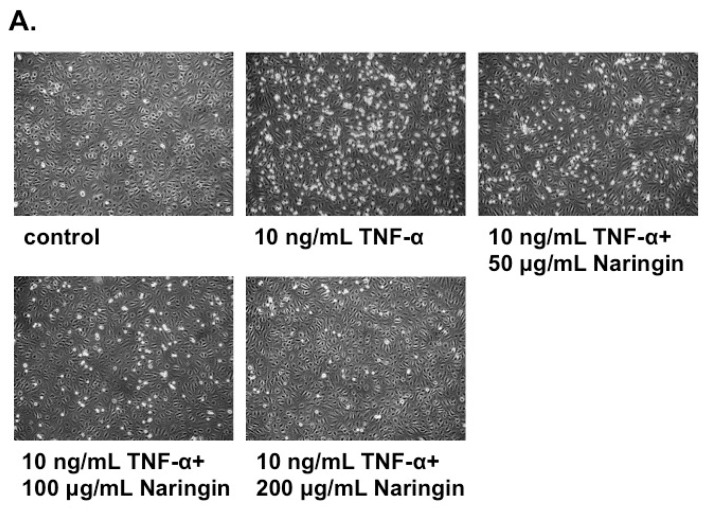

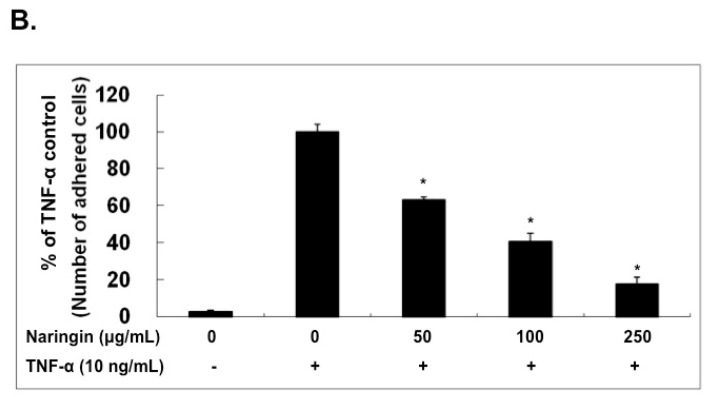
Naringin inhibited the adhesion of leukemic monocytes (THP-1) to TNF-α-stimulated human umbilical vein endothelial cells (HUVECs). (**A**) HUVECs were stimulated with TNF-α (10 ng/mL) for 6 h with/without Naringin (50, 100 and 200 mg/mL) pretreatment for 18 h. The adhesion of THP-1 cells to HUVECs was carried out for 30 min at 37 °C. Microphotographs (100×) showing the adhesion of THP-1 to TNF-α-stimulated HUVECs under conditions as indicated; (**B**) The number of THP-1 cells adhered to TNF-α-stimulated HUVECs was counted and compared. (Significance compared with positive control of TNF-α treatment only, * *p* < 0.05). Values were mean ± SEM of three independent tests.

To examine the effect of Naringin on the monocyte-endothelium adhesion, HUVECs were treated with or without the indicated concentration of naringin (50, 100 and 200 μg/mL) for 18 h and followed with TNF-α (10 ng/mL) for 6 h. THP-1 cells were added to the HUVECs culture to observe the adhesion activity. There was less than 10 percents of THP-1 cells adherent to HUVECs without TNF-α-stimulation. However, TNF-α greatly stimulated the adhesion of THP-1 cells to HUVECs. Naringin inhibited the TNF-α-induced adhesion of THP-1 cells to HUVECs dose-dependently, as shown in Figure 2A. Naringin at the concentration of 100 μg/mL approximately reduced more than half of the adhesion compared to TNF-α control without naringin pretreatment (Figure 2B).

### 2.3. Effect of Naringin on mRNA Expression and Protein Levels of ICAM-1, VCAM-1, and E-Selectin

**Figure 3:**
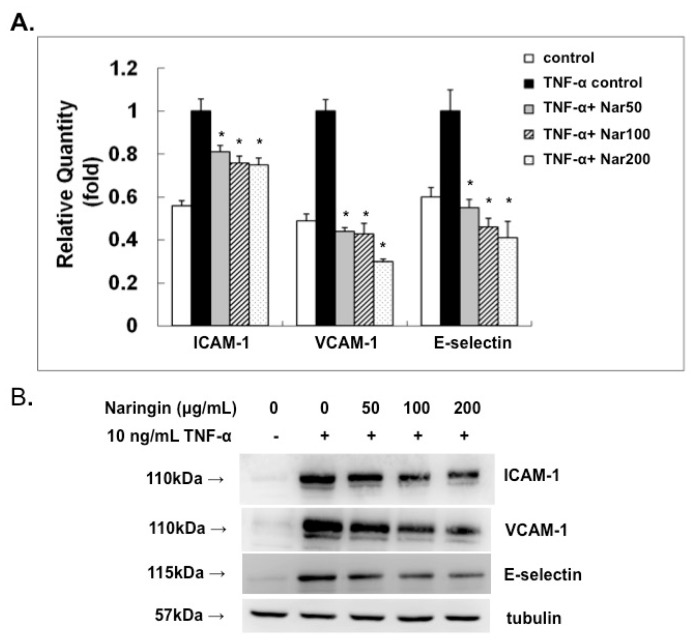

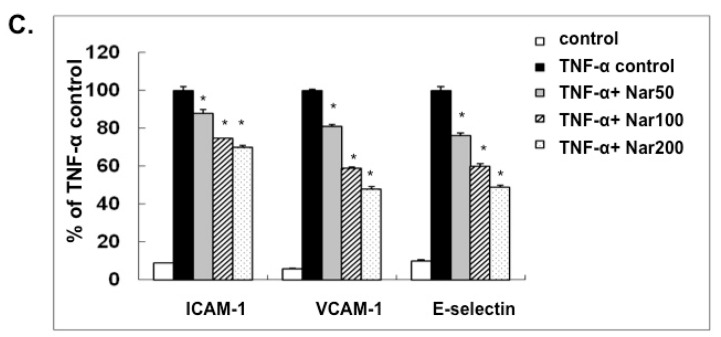
Effect of Naringin on the mRNA and protein expressions of vascular cell adhesion molecule-1 (VCAM-1), intracellular adhesion molecule-1 (ICAM-1), and E-selectin in TNF-α-stimulated human umbilical vein endothelial cells (HUVECs). HUVECs were stimulated with TNF-α (10 ng/mL) for 6 h with/without Naringin (50, 100 and 200 mg/mL) pretreatment for 18 h. (**A**) RNA was isolated for qRT-PCR analysis; (**B**) Cell extracts were subjected to 12% SDS-PAGE and Western blot analysis. Tubulin was used as an internal control; (**C**) Densitometric analysis of the protein levels in (**B**). Data were quantified and are presented as mean values ± SD of three independent experiments. Analysis of variance was used to compare the multiple group means, followed by Newman–Keuls test (significance compared with positive control of TNF-α treatment only, * *p* < 0.05).

In order to further understand the potential mechanism of the inhibitory effect of naringin on anti-atherosclerosis, we analyzed the effect of naringin on the mRNA expression of VACM-1, ICAM-1, and E-selectin by real time PCR method and Western blot. In the present study, the expression of cell adhesion molecules was therefore analyzed at 6 h after TNF-α-stimulation. As shown in Figure 3, the mRNA and protein expression levels of VCAM-1, ICAM-1, and E-selectin were markedly increased by TNF-α-stimulation for 6 h in HUVECs. The inhibitory effect of naringin pretreatment (50, 100 and 200 μg/mL) for 18 h on the TNF-α-induced expressions of VCAM-1, ICAM-1 and E-selectin at both the mRNA and protein levels was noticed.

### 2.4. Effect of Naringin on the Expression of Fractalkine, MCP-1, and RANTES in TNF-α-Stimulated HUVECs

**Figure 4:**
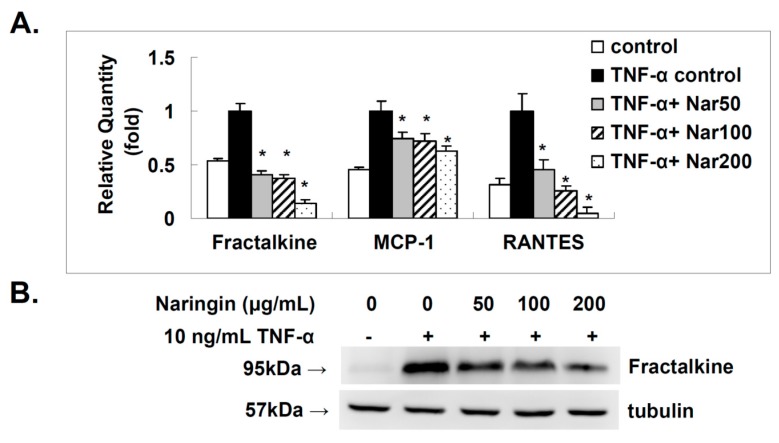

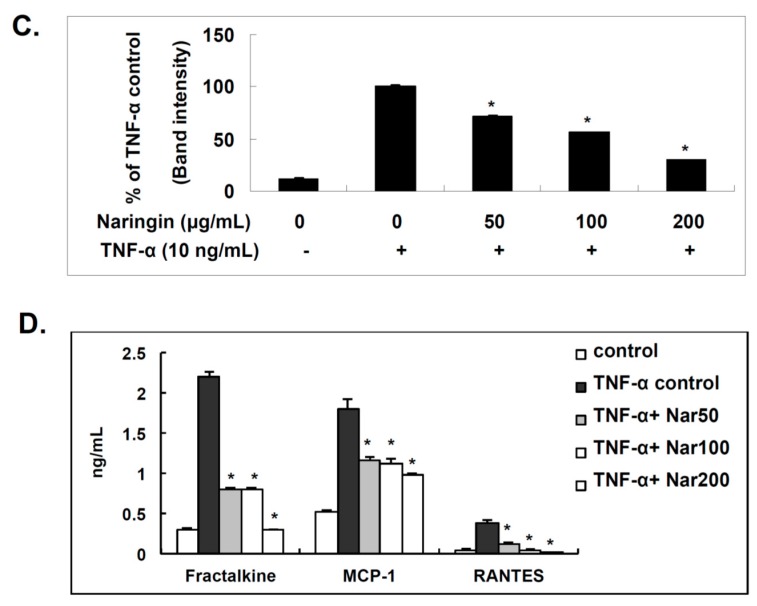
Effect of Naringin on the mRNA and protein expression levels of fractalkine, monocyte chemotactic protein-1 (MCP-1) and regulated on activation of normal T cell expressed and secreted (RANTES) in TNF-α-stimulated human umbilical vein endothelial cells (HUVECs). HUVECs were stimulated with TNF-α (10 ng/mL) for 6 h with/without Naringin (50, 100 and 200 mg/mL) pretreatment for 18 h. (**A**) RNA was isolated for qRT-PCR analysis. The effect of Naringin on mRNA expression of fractalkine, MCP-1 and RANTES; (**B**) Cell extracts for fractalkine were subjected to 12% SDS-PAGE and Western blot analysis. Tubulin was used as an internal control. Results from densitometric analysis of protein levels in (**C**) are shown below the representative data; (**D**) The levels of soluble fractalkine, MCP-1 and RANTES in conditioned media were determined by ELISA. Data were quantified and are presented as mean values ± SD of three independent experiments. Analysis of variance was used to compare the multiple group means, followed by Newman-Keuls test (significance compared with positive control of TNF-α treatment only, * *p* < 0.05).

Fractalkine, MCP-1, and RANTES are documented to exert pro-atherogenic activity since they contribute to the recruitment of inflammatory T cells and macrophages into atherosclerotic lesions [19]. The deletions of MCP-1, CCR2 or fractalkine receptor (CX3CR1) individually resulted in a 50% decrease in atherosclerotic lesion [20,21,22]. The expressions of fractalkine, MCP-1, and RANTES at the mRNA level in HUVECs were investigated. Stimulation of HUVECs with TNF-α (10 ng/mL) for 6 h resulted in a significantly increased expressions at mRNA level (Figure 4A) that were suppressed in a dose dependent manner (Figure 4A) by pretreatment with naringin. Cellular expression of fractalkine, as determined by Western blot (Figure 4B,C), was induced by TNF-α (10 ng/mL) and dose-dependently inhibited by naringin. ELISA was used to determine the levels of soluble chemokines. As shown in Figure 4D, the induced levels of soluble fractalkine, MCP-1, and RANTES in the conditioned media collected from TNF-α-stimulated HUVECs were decreased by the pretreatment with indicated concentrations of naringin dose-dependently. We discovered that naringin could indeed down-regulate the TNF-α induced expression of fractalkine, MCP-1, and RANTES in HUVECs.

### 2.5. Naringin Inhibited Nuclear Translocation of NF-κB Stimulated by TNF-α

**Figure 5:**
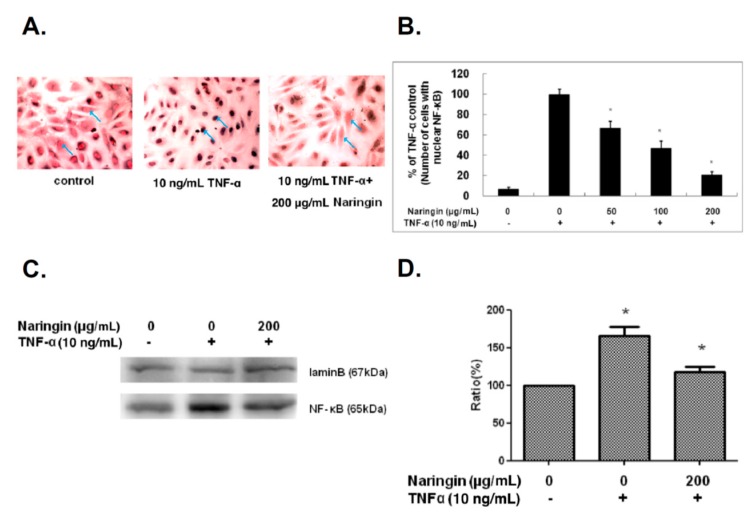
Effect of Naringin on the nuclear translocation of NF-κB in TNF-α-treated human umbilical vein endothelial cells (HUVECs). HUVECs were stimulated with TNF-α (10 ng/mL) for 1 h with/without Naringin (50, 100 and 200 mg/mL) pretreatment for 18 h. NF-κB localization was visualized by immunocytochemical method. (**A**) Representative microphotographs (200 μg/mL) were acquired by light microscope; (**B**) The numbers of positive signals (dark blue) in the cell nuclei were determined and compared; (**C**) Cell nuclear extracts for NF-κB were subjected to 12% SDS-PAGE and Western blot analysis. Lamin B was used as an internal control. Results from densitometric analysis of protein levels in (**D**) are shown below the representative data. Data were quantified and are presented as mean values ± SD of three independent experiments (significance compared with positive control of TNF-α treatment only, * *p* < 0.05).

NF-κB regulates various inflammatory and immune responses. NF-κB is activated by TNF-α in HUVECs and rapidly translocates into the nucleus, consequently promoting the transcription of diverse genes encoding cytokines, growth factors, cell adhesion molecules, and pro-/antiapoptotic proteins [23,24]. Therefore, the nuclear translocation of NF-κB was used in the present study to evaluate its activity. As shown in Figure 5A, the TNF-α-induced translocation of NF-κB with color developed by the reaction of peroxidase with ImmPACT™ SG substrate (VECTOR) and counterstained with nuclear fast red (VECTOR) was demonstrated by the dark blue colored nuclei, which was then significantly inhibited by treatment with naringin. These results demonstrated that naringin inhibited TNF-α-induced translocation of NF-κB in a dose-dependent manner (Figure 5B). Naringin treatment at 200 μg also showed the reduced protein level of NF-κB in nucleus after 24 h incubation compared to positive control with TNF-α stimulation (Figure 5C,D).

### 2.6. Inhibitory Effect of Naringin on the Activation of IKK/NF-κB Pathway Stimulated by TNF-α

**Figure 6:**
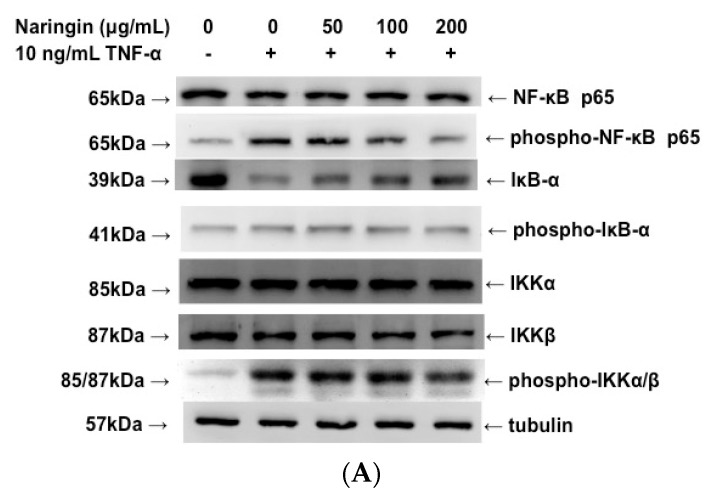

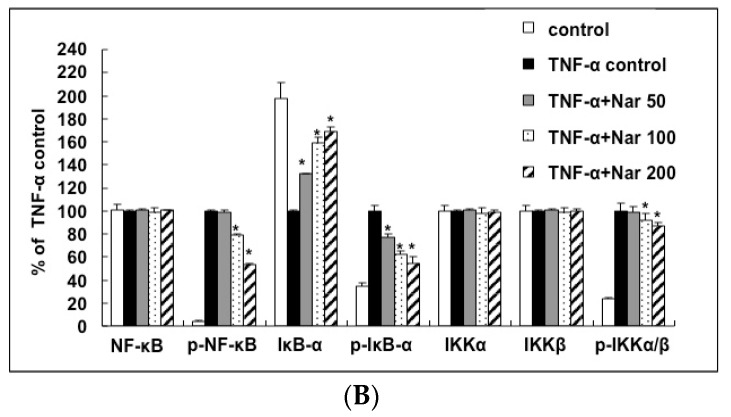
Effect of Naringin on the activation of IKK/NF-κB pathway in TNF-α-stimulated human umbilical vein endothelial cells (HUVECs). HUVECs were stimulated with TNF-α (10 ng/mL) for 1 h with/without Naringin (50, 100 and 200 mg/mL) pretreatment for 18 h. (**A**) Cell extracts were subjected to 12% SDS-PAGE. Western blotting for the native or phosphorylated forms of NF-κB, IκB-α and IKK α/β was performed. Tubulin was used as a loading control; (**B**) Densitometric analysis of the protein levels in (**A**). Data were quantified and are presented as mean values ± SD of three independent experiments (significance compared with positive control of TNF-α treatment only, * *p* < 0.05).

NF-κB is activated due to reduced phosphorylation and degradation of IκBα, and the nuclear translocation of NF-κB/p65 is preceded by IκBα. We would like to find out whether naringin could inhibit the phosphorylation and degradation of IκBα, and thereby inhibit the nuclear translocation of NF-κB/p65. As shown in Figure 6, TNF-α significantly activated the phosphorylation of IKKα/β, IκB-α and NF-κB itself. Pretreating HUVECs with different concentrations of naringin for 18 h dose-dependently suppressed the phosphorylation of these TNF-α-activated proteins in the IKK/NF-κB pathway. It is known that the phosphorylation of IκB-α could lead to the degradation of this protein, and the suppressed phosphorylation of IκB-α by naringin led to the increase or less degradation of IκB-α in TNF-α-stimulated HUVECs.

## 3. Discussion

The vascular endothelial layer plays an important role in regulating blood flow and preventing coagulation. It influences the underlying vascular homeostasis, including regulation of the recruitment of circulating leukocytes and their transendothelial migration, and also activates the innate immune system to control inflammation. Breakdown of the vascular endothelium results in uncontrolled inflammatory stimuli and tissue damage. The formation of atherosclerotic plaque is associated with activation of the pro-inflammatory gene transcriptional program in vascular endothelial cells when challenged by any tissue damage in response to traumatic, infectious, post-ischemic, toxic, or autoimmune injury [25,26]. Therefore, in this study, we used HUVECs as a model to characterize the impact of naringin on TNF-α-induced inflammation related to the expression of chemokines and adhesion molecules.

Naringin has been shown to possess anti-inflammatory and anti-angiogenic effects [27]. The anti-inflammatory effect of naringin may be due to the scavenging activity of reactive oxygen species (ROS), down-regulation of pro-inflammatory mediators, and suppression of the ROS system [28,29]. Naringin supplementation inhibited plaque formation in wild-type mice fed the high-fat/high-cholesterol diet to limit atherosclerosis through down-regulation of monocyte adhesion to endothelial cells and smooth muscle cell proliferation [30]. Naringin treatment has been reported to block PI3K/AKT/mTOR/p70S6K pathway induced by TNF-α and Ras/Raf/ERK pathway participated in p21WAF1 induction of vascular smooth muscle cells (VSMCs) [30,31,32]. As for the impact of naringin on HUVECs relevant to the atherosclerosis, it is not clear so far.

Leukocyte recruitment into sites of inflammation is a multi-step process that leads to the accumulation of cells in the inflammatory tissue. Pro-inflammatory chemokines such as fractalkine, MCP-1 and RANTES are produced by immunological cells and cells of the cardiovascular system. These chemokines contribute to the chemo-attraction of T cells and monocytes and promote strong adhesion of leukocytes to activated early atherosclerotic lesions [19]. In more advanced plaques, chemokines are also crucial for foam cell persistence and the production of numerous chemotactic factors, other cytokines, and cell adhesion molecules that worsen the inflammatory reactions. In this study, naringin exhibited an anti-atherosclerotic effect associated with down-regulation of mRNA and protein expression of fractalkine, MCP-1, and RANTES that stimulated by TNF-α. In addition to pro-inflammatory chemokines, the adhesion molecules including ICAM-1, VCAM-1, and E-selectin play a key role in the adherence and infiltration of leukocytes to endothelial cells, maintaining chronic inflammation in the cardiovascular system [33,34,35]. Also, these adhesion molecules are up-regulated in the vascular endothelium of CVD patients and are proposed as biomarkers and predictors for cardiovascular risk [34,36,37]. In the present study, Naringin not only suppressed the release of chemokines such as fractalkine, MCP-1, and RANTES but also inhibited the mRNA and protein expression of TNF-α-induced ICAM-1, VCAM-1, and E-selectin in HUVECs. Previously, antibody blockade of ICAM-1 and VCAM-1 was shown to ameliorate inflammation in an experimental animal model of the atherogenic process [17,18]. Altogether, it indicated that the naringin might be beneficial for the treatment of CVD.

**Figure 7:**
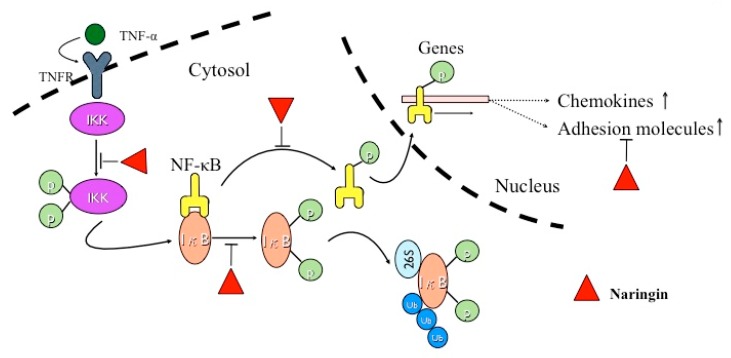
Schematic diagram illustrates the molecular mechanism that is responsible for the anti-atherosclerotic effect of Naringin.

NF-κB is a transcription factor that is involved in innate immune and inflammatory responses. NF-κB is activated significantly with the severity of atherosclerosis or vascular inflammation in experimental animal models and in patients with CVD [9,38]. Under stable-state conditions, NF-κB is present in the cytoplasm and bound to its naturally occurring IκB. The activation of NF-κB by extracellular stimuli such as TNF-α is initiated by the activation of IκB kinase (IKK). The activated IKK phosphorylate IκB triggers ubiquitinylation and subsequent degradation of IκB. The disengagement of IκB releases NF-κB from an inactive complex, which then translocates from cytoplasm to nucleus, where it binds to κB-specific promotor/enhancer regions of target genes [39,40]. NF-κB may be one of the prime targets for novel anti-atherosclerotic therapies. In our study, naringin inhibited TNF-α-induced IκBα phosphorylation/degradation, nuclear translocation of NF-κB/p65, and IKKα/β phosphorylation in HUVECs. In other words, naringin suppressed the activation of NF-κB through suppressing IKK activity and subsequent IκB phosphorylation and degradation. ICAM-1, VCAM-1, E-selectin, MCP-1, RANTES and fractalkine gene expressions are regulated by the transcription factor NF-κB, which also controls the transcription of a diverse array of pro-inflammatory genes [41,42]. A recent report has also shown that the expressions of ICAM-1, V-CAM-1 and E-selectin could be inhibited by suppressing NF-κB signaling pathway [43]. In our study, IκB, an NF-κB inhibitor, suppressed the TNF-α-induced ICAM-1, VCAM-1, E-selectin, MCP-1, RANTES and fractalkine mRNA/protein accumulations in HUVECs. Therefore, naringin promoted IκB-α to maintain the inhibition of NF-κB, and inhibited the expression of adhesion molecules and chemokines by blockage of NF-κB activation in HUVECs. The net effects of naringin exhibited in the present study are illustrated (Figure 7).

In comparison with the present literature, Li *et al.*, studied the anti-oxidative and anti-inflammatory effects of naringin on TNF-α induced HUVECs revealed that naringin not only inhibited TNF-α induced mRNA and protein expressions of ICAM-1 and VCAM-1 but also suppressed NF-κB through PI3K/Akt signaling pathways [44]. This finding is in line with a previous experiment on the inhibition effect of naringin in MMP-9 expression, which also suggested that the anti-atherosclerotic effect of naringin was through the PI3K/AKT/mTOR/p70S6K pathway [31]. Naringin also successfully inhibited cisplatin induced oxidative stress and inflammatory response. It was hypothesized that naringin was responsible for the inhibition of TNF-α cytokines levels and pro-inflammatory mediator expression [45]. Another study demonstrated that naringin can rescue TNF-α induced inhibition of osteogenesis on bone marrow-derived mesenchymal stem cells, which indicated naringin decreased expressions of p-IκBα and nuclear p65, and thus repressed NF-кB pathway activated by TNF-α treatment [46]. Based on the above studies, our study corresponded not only to provide an evidence of naringin in suppression of adhesion molecules and pro-inflammatory chemokines, but also revealed the underlying mechanism of naringin through the phosphorylated NFκB/IκB signaling pathway.

As an important step in the early stage of atherosclerosis, the monocyte-endothelial cell interaction has been extensively studied. In the present study, we demonstrated that naringin could inhibit the monocyte adhesion induced by TNF-α. Previous studies have shown that naringin could also inhibit the monocyte adhesion induced by high glucose. However, naringin did not have any effect on the monocyte adhesion induced by oxLDL, even though naringin could inhibit the oxidation of LDL [27], suggesting a different signal pathway was involved in the process. In a recent paper, naringin was shown to reduce the expressions of ICAM-1 and VCAM-1 induced by TNF-α and it was proposed that a similar pathway was involved [17,18]. However, our study for the first time demonstrated that the anti-atherosclerotic effect of naringin was mediated by thoroughly suppressing the expression of chemokines, including MCP-1 RANTES and fractalkine, and cell adhesion molecules, including ICAM-1, VCAM-1 and E-selectin. The inclusive effect of naringin on inhibiting both chemokines and cell adhesion molecules further provided a mechanistic explanation for its effect on preventing atherosclerosis.

The present study provides new insight into the molecular mechanisms related to the anti-atherosclerotic effect of naringin on HUVECs. It is a model system for the study of the regulation of endothelial cell function and can be treated as the role of endothelium in response to the blood vessel wall with shearing forces and the development of atherosclerotic plaques. We confirmed the anti-atherosclerotic effect of naringin through NF-кB pathway in an *in vitro* study; however, further *in vivo* studies are needed as well. The findings of this study, in part, explain the possible therapeutic or preventive effects of naringin in the treatment of atherosclerosis.

## 4. Materials and Methods

### 4.1. Cell Culture

HUVECs were isolated from the veins of human umbilical cords and grown in EGM provided by Clonetics (Walkersville, MD, USA). Cells were maintained in a humidified atmosphere with 5% CO_2_/95% air at 37 °C and passaged 3–5 times prior to use in experiments. To examine the effect of naringin on cell function, cells at 80%–90% confluency were treated with 0–200 μg/mL of naringin for 24 h. The Institutional Review Board of Chang-Gung Memorial Hospital approved the experimental use of HUVECs, and informed consent was obtained from each donor before experiments commenced.

### 4.2. MTT Assay

Cells with (50, 100, or 200 μg/mL) or without naringin treatment were washed once with PBS, followed by the addition of 1 mL DMEM containing 0.05 mg/mL 3-(4,5-dimethylthiazol-2-yl)-2,5-diphenyltetrazolium bromide (MTT). After incubation at 37 °C for 1 h, the media were removed and the formazan crystals in the cells were solubilized in 1 mL DMSO for optical density (OD) reading at 570 nm using a spectrophotometer.

### 4.3. LDH Assay

Cells were seeded in 200 μL of culture medium with or without naringin, followed by the addition of 2 μL of lactate dehydrogenase (LDH). After incubation at 37 °C for 24 h, the media were stopped and 50 μL of supernatant was collected. LDH activity was measured using the CytoTox 96^®^ Non-Radioactive Cytotoxicity Assay (Promega, Mannheim, Germany). Disruption of plasma membrane integrity leads to a release of LDH into the supernatant and results in the conversion of a tetrazolium salt into a red formazan product and read at 490 nm spectrophotometrically.

### 4.4. Monocyte Adhesion Assay

Monolayers of HUVECs were pretreated with or without naringin for 18 h, followed by induction with TNF-α for 6 h. HUVECs were then incubated with 2 × 10^5^ THP-1 cells (monocyte, ATCC, Manassas, VA, USA), for 30 min in a humidified atmosphere with 5% CO_2_/95% air at 37 °C. After incubation, non-adherent cells were removed by washing with PBS twice. In total, six random high-power microscopic fields (HPF) (100×) were photographed and the numbers of adhesion cells were directly counted.

### 4.5. RNA Isolation and Real-Time Polymerase Chain Reaction

Total cellular RNA was isolated by cell lysis in a guanidinium isothiocyanate buffer, followed by a single step phenol–chloroform–isoamyl alcohol extraction procedure modified from that previously described [47]. Briefly, untreated or treated cells with Naringin were harvested and lysed in 4 M guanidinium isothiocyanate, 25 mM sodium citrate (pH 7.0), 0.5% sodium sarkosine and 0.1 M β-mercaptoethanol. Sequentially, 1/10 volume of 2 M sodium acetate (pH 4.0), one volume of phenol and 1/5 volume of chloroform–isoamyl alcohol (49:1, *v*:*v*) were added to the homogenate. After vigorous vortexing for 30 s, the solution was centrifuged at 10,000× *g* for 15 min at 4 °C. After removal of the aqueous phase, RNA was precipitated by the addition of 0.5 mL isopropanol. For real-time PCR analysis, reverse transcription was performed using 1 μg of total RNA and oligo (dT) primers in a 20 μL reaction according to the manufacture’s protocol (PE Applied Biosystems, Foster City, CA, USA). Real-time PCR was performed using the Mx3005 QPCR system (Strategene, La Jolla, CA, USA) with SYBR green (Applied Biosystems) as a dsDNA-specific binding dye. The PCR was cycled 40 times after initial denaturation (95 °C, 2 min) with the following parameters: denaturation, 95 °C, 15 s; and annealing and extension, 60 °C, 1 min. The threshold cycle was recorded for each sample to reflect the mRNA expression level. Sequences for the specific primers used in the PCR are VCAM-1 forward primer (5′-CATGACCTGTTCCAGCGAGG-3′) and reverse primer (5′-CATTCACGAGGCCACCACTC-3′); ICAM-1 forward primer (5′-GCAAGAAGATAGCCAACCA-3′) and reverse primer (5′-TGCCAGTTCCACCCGTTC-3′); E-selectin forward primer (5′-GGTTG AGTGTGATGCTGTGA-3′) and reverse primer (5′-GAAGGTGAACTCTCCAGCAG-3′); MCP-1 forward primer (5′-GACCACCTGGACAAGCAAAC-3′) and reverse primer (5′-GTCTGGGGAAAGCTAGGGGA-3′); RANTES forward primer (5′-TTCCTGTATGACTCCCGGCT-3′) and reverse primer (5′-CTTCTCTGGGTTGGCACACA-3′); Fractalkine forward primer (5′-ATCAACAGAACCAGGCATCA-3′) and reverse primer (5′-GCCGCCATTTCGAGTTAG-3′); and GAPDH forward primer (5′-GACCTGACCTGCCGTCTA-3′) and reverse primer (5′-AGGAGTGGGTGTCGCTGT-3′).

### 4.6. Protein Extraction and Western Blot Analysis

HUVECs were treated with 0, 50, 100 and 200 μg naringin for 24 h, and then rinsed twice with ice-cold PBS and harvested in a lysis buffer followed by sonication. Nuclear and cytoplasmic proteins were extracted with the Nuclear and Cytoplasmic Extraction Reagents (Beyotime, Jiangsu, China) according to the manufacturer’s instructions. Protein concentrations were determined by the Bradford method (Bio-Rad, Hercules, CA, USA). Samples with equal amount of proteins were subjected to 10% SDS-PAGE and transferred onto a polyvinylidene difluoride (PVDF) (Millipore, Bedford, MA, USA) membrane. The membrane was incubated at room temperature in blocking solution (1% BSA (bovine serum albumin), 1% goat serum in PBS for 1 h, followed by 2 h incubation in blocking solution containing an appropriate dilution (1:1000) of primary antibody, e.g., anti-VCAM-1, anti-ICAM-1, anti-E-selectin, anti-fractalkine (CX3CL1), anti-tubulin antibody (NeoMarkers, Fremonk, CA, USA), anti-phosphorylated IKK α/β, anti-total IKK-α, anti-total IKK-β, anti-phosphorylated p65 NF-κB, anti-total p65 NF-κB, anti-phosphorylated IκB-α, anti-total IκB-α (Cell Signaling, Boston, MA, USA) and anti-lamin B (Santa Cruz Biotechnology, Inc., Dallas, TX, USA). After washing, the membrane was incubated in PBS containing goat anti-mouse IgG conjugated with horseradish peroxidase (Sigma, St. Louis, MO, USA) for 1 h. The membrane was washed and the positive signals were developed with chemiluminescence reagent (Amersham Pharmacia Biotech, Little Chalfont Buckinghamshire, UK). The membrane was then exposed to Fuji medical X-ray film (Fuji Ltd., Tokyo, Japan) for 3 min.

### 4.7. Fractalkine, MCP-1, and RANTES ELISA

The levels of fractalkine, monocyte chemotactic protein-1 (MCP-1), and regulated upon activation normal T cell expressed and presumably secreted (RANTES) in conditioned media collected from HUVECs were determined by a human specific ELISA kit (R & D Systems, Minneapolis, MN, USA). The experimental steps were carried out as described in the protocol provided by the manufacturer. This process was performed three times, and the results of each time were analyzed in duplicate.

### 4.8. Immunocytochemical Staining of NF-κB in HUVECs

Cells grown on coverslips were fixed in 4% formaldehyde (pH 7.5) for 15 min at room temperature and immersed in blocking solution containing 1% BSA and 1% goat serum in PBS for 30 min followed by the incubation with 50× dilution of monoclonal antibody against NF-κB p65 (Santa Cruz Biotechnology, Inc., Dallas, TX, USA) in blocking solution for 60 min. After washing, cells were incubated in PBS containing biotinylated goat anti-rabbit IgG for 15 min followed by washing and incubated with streptavidin conjugated with horseradish peroxidase (Thermo, Scientific, Waltham, MA, USA) for 10 min. Cells were washed and the positive signals were developed with ImmPACT™ SG Peroxidase Substrate for 15 min. After counter-staining with nuclear fast red (Vector Labs, Inc., Burlingame, CA, USA) and washing, cells were mounted and analyzed by light microscope.

### 4.9. Statistical Analysis

All statistical analyses were performed using SigmaStat statistical software (version 2.0, Jandel Scientific, San Rafael, CA, USA). Results were represented as means ± standard deviation (SD). ANOVA was carried out when multiple comparisons were evaluated. Values were considered to be significant at p values less than 0.05. All experiments were independently performed at least three times, and the analysis of each experiment was carried out in triplicate at the same time.

## 5. Conclusions

This study demonstrated that naringin inhibited the expressions of TNF-α-induced adhesion molecules and chemokines to decrease the adhesion of THP-1 to TNF-α-stimulated HUVECs through blocking the NF-κB translocation and phosphorylation of NF-κB and IκB signaling pathways. These findings may provide a theoretical basis of the clinical application of naringin for atherosclerosis diseases.
